# Social Determinants of Depression: The Intersections of Race, Gender, and Socioeconomic Status

**DOI:** 10.3390/brainsci7120156

**Published:** 2017-11-24

**Authors:** Shervin Assari

**Affiliations:** 1 Center for Research on Ethnicity, Culture, and Health (CRECH), School of Public Health, University of Michigan, Ann Arbor, MI 48109, USA; assari@umich.edu; Tel.: +1-734-232-0445; Fax: +1-734-763-9265; 2 Department of Psychiatry, University of Michigan, Ann Arbor, MI 48109, USA; 3 Institute for Healthcare Policy and Innovation (IHPI), University of Michigan, Ann Arbor, MI 48109, USA

**Keywords:** race, ethnicity, ethnic groups, blacks, African Americans, gender, depression, socioeconomic status (SES), income, social class

## Abstract

Background: Despite the wealth of literature on social determinants of mental health, less is known about the intersection of these determinants. Using a nationally representative sample, this study aimed to study separate, additive, and multiplicative effects of race, gender, and SES on the risk of major depressive episode (MDE) among American adults. Methods: National Survey of American Life (NSAL) included 3570 African Americans and 891 Whites. Race, gender, socioeconomic status (SES, household income, education, employment, and marital status) were independent variables. Twelve-month MDE was measured by the Composite International Diagnostic Interview (CIDI). A series of logistic regressions were used to analyze the data. Results: In the pooled sample, race and household income, but not gender, education, employment, and marital status were associated with 12-month MDE. Gender interacted with the effects of income on MDE, suggesting that the association between household income and MDE is larger for women than men. In race by gender specific models that controlled for other SES indicators, high income was protective for White women, education was protective for African American women, and high income became a risk factor for African American men. High income did not show a risk effect for African American men in the absence of other SES indicators. Conclusions: Findings suggest that race, gender, and class interact on how SES indicators, such as education or income, become a protective or a risk factor for MDE among American Adults. When the outcome is MDE, White women benefit more from income, African American women gain from education, however, the residual effect of high income (above and beyond education, employment, and marital status) may become a risk factor for African American men.

## 1. Background

Most available theories and frameworks on social determinants of health (SDH) have assumed that a high socioeconomic status (SES) is universally protective across populations [1]. Fundamental Cause Theory, developed by Link and Phelan in 1996, emphasizes the salience of the high SES for physical and mental health of populations [1]. In the same line, Mirowsky and Ross have described the protective effect of SES on health as “enduring, consistent, and growing” [2]. Marmot has also argued about the social gradient in health, meaning that people with the highest SES enjoy the highest level of health, and people with the lowest levels of SES suffer the worst health [3,4,5].

Most of the empirical work in sociology and epidemiology has also focused on the overall effects of SES on health [6,7,8]. Such protective effects of SES indicators in overall samples are documented by multiple state-of-the-art studies, such as the British Cohort Study (BCS) [9], the Survey of Health, Aging and Retirement in Europe (SHARE) [10], the Health and Retirement Study (HRS) [11], the Americans’ Changing Lives Study [10,12,13], the French GAZEL cohort [14,15], and the Panel Study of Income Dynamics [16].

Populations, however, differ in their abilities to translate their SES resources to health outcomes [17,18]. Aside from the mainstream literature that has traditionally focused on the overall effects of SES [1,19,20,21], a growing body of research shows that the effects of SES indicators on health outcomes may depend on race and gender [17,18,22]. Although some evidence has documented cross-population variations in the effects of SES on health [23], more research is needed on the interactive effects of race, gender, and class on population health.

A number of theoretical and empirical evidence suggest that the effects of SES may depend on race. Double Jeopardy [24], Triple Jeopardy [25], Multiple Jeopardy [26], Multiple Disadvantage [27], and Cumulative Disadvantage [28] theories all conceptualize minority populations as a vulnerability status. According to these theories, African Americans are expected to be more susceptible to the presence or absence of SES indicators [26]. In contrast to these frameworks, Blacks’ diminished return framework has suggested that possibly due to structural racism [29,30,31], health gains from high SES are systemically larger for Whites than African Americans [17,18]. Not only SES indicators may result in smaller health gain for African Americans [17,18], possibly due to discrimination [32] and the high costs of upward social mobility [33,34], high SES may even be associated with worse mental health outcomes for African Americans, particularly African American males [3,23,35,36]. To give an example, a national study found a positive association between family income and risk of depression among African American boys [23].

To gain a better understanding of the complexities in the effects of race, gender, and SES on mental health in the United States (US), this study was conducted to investigate the separate, additive, and multiplicative effects of race, gender, and SES on major depressive episode (MDE) in a national sample of American Adults.

## 2. Methods

### 2.1. Design, Participants, and Sampling

Data from the National Survey of American Life (NSAL) were used for this study. Funded by the National Institute of Mental Health (NIMH) and a part of the Collaborative Psychiatric Epidemiology Surveys (CPES), NSAL was conducted by University of Michigan [37,38]. More information regarding the NSAL sampling is available elsewhere [38].

NSAL enrolled a national household probability sample of Black adults that are representative to US. The NSAL included 3570 African American and 891 non-Hispanic White individuals aged 18 or older. NSAL sampled individuals from Americans who were residing in the US at the time of the study. Although the NSAL has also included 1621 Caribbean Blacks, this study did not include them in the current analysis. The reason behind this decision was that Caribbean Blacks are a unique ethnic group of Blacks with high SES, more recent immigration, and without history of slavery in the United States. In addition, most of the theory and literature on unequal gain of equal resources [18], also known as Blacks’ diminished return [17], is on African Americans, not Caribbean Blacks. Whites and African American sample was enrolled from large cities or other urban and rural areas. 

### 2.2. Ethics

The NSAL study protocol received Institute Review Board (IRB) approval by the University of Michigan, Ann Arbor (B03-00004038-R1). Respondents received financial incentive as compensation for their participation. Informed consent was obtained from all participants included in the study. All procedures performed in studies involving human participants were in accordance with the ethical standards of the institutional and/or national research committee and with the 1964 Helsinki Declaration and its later amendments or comparable ethical standards.

### 2.3. Interviews

All the interviews were performed in English. Face-to-face interviews were performed using computer-assisted personal interviews (CAPI), where the respondent uses a computer to answer the questions. CAPI is believed to improve the quality of the data collection when the questionnaire is long and complex [39]. The response rate was 70.7% for African Americans and 69.7% for non-Hispanic Whites.

### 2.4. Measures

The current analysis included data on demographics (age, gender, race, ethnicity), SES (household income, education, employment, and marital status), and 12-month MDE. Age was treated as a continuous variable. Gender was female versus male [reference category].

### 2.5. Ethnicity

Participants’ race and ethnicity were self-identified by the individuals. African Americans were those who identified as Black but did not have ancestral ties to the Caribbean. Ethnicity was treated as a dichotomous variable, with Whites as the reference group.

### 2.6. SES

For SES indicators, the following were measured: household income, education, employment, and marital status. All SES indicators were measured using self-reported data, via interview. Household income was conceptualized as a continuous measure (income in dollars divided by 1000). Education (0–11 years [reference category] vs. 12 years or more), employment (currently employed [reference category] vs. all other categories), and marital status (currently married vs. all other categories [reference category]) were all treated as dichotomous variables.

### 2.7. Depression

12-month MDE was measured using the World Mental Health (WHO) Composite International Diagnostic Interview (CIDI). CIDI is a fully structured diagnostic interview schedule that is implemented by lay (non-clinician) interviewer who have received training. Originally developed for the WHO project initiated in 2000 [40], the CIDI makes the diagnoses based on Diagnostic and Statistical Manual, Fourth Edition (DSM-IV) criteria. This interview schedule determines who endorses criteria of lifetime and recent disorders based on DSM-IV-TR and the International Classification of Diseases-10 (ICD-10) [41]. Clinical reappraisal studies have shown that CIDI based-diagnoses have good concordance with blinded clinical diagnoses [40,41,42,43], particularly for MDE [42]. This measure provides valid findings for African Americans [44,45,46].

### 2.8. Statistical Analysis

To accommodate the complex survey design of the NSAL, data were analyzed using Stata 13.0 (Stata Corp., College Station, TX, USA). Taylor series approximation technique was used to estimate the complex design-based standard errors and variance. Thus, all of the reported standard errors and confidence intervals reflect the study’s complex design. All of the percentages reported in this study are weighted, and thus represent proportions to the nation. Adjusted odds ratio (OR), 95% confidence interval (CI), and *p* values were reported. *p* values of less than 0.05 were considered statistically significant.

Several survey logistic regression models were used for multivariable analysis. The overall *p* values were significant for all of the logistic models. In these models, SES indicators were the independent variables, MDE was the dependent variable, and age and region were covariates. The associations of interest were estimated in the pooled sample and then based on race by gender groups. First, models tested the additive effects of SES indicators. Then, models tested the multiplicative effects of race, gender, and SES.

## 3. Results

### 3.1. Descriptive Statistics

Table 1 describes age, household income, education, employment, marital status, and 12-month MDE in the pooled sample and based on the race. Income, education, being employed, and being married were higher for Whites than African Americans.

### 3.2. Multivariable Models in the Pooled Sample to Test Separate Effects

Table 2 summarizes the results of two logistic regressions in the pooled sample, with MDE as the outcome and income as the predictor. In *Model 1* that only included main effects, higher household income was associated with lower odds of MDE in the pooled sample (OR = 0.92, 95% CI = 0.84–1.00). In *Model 2*, which also included income by gender and income by race interaction terms, an interaction was found between gender and household income on MDE (OR = 0.87, 95% CI = 0.76–1.00), suggesting that the protective effect of household income on MDE is significantly different between men and women (Table 2).

Table 3 summarizes the results of two logistic regressions in the pooled sample, with MDE as the outcome and education 12 years or more as the predictor. In *Model 1* that only included main effects, education was not associated with odds of MDE in the pooled sample (*p* > 0.05). *Model 2* that also included education by gender and education by race interactions did not show any interaction between gender and education and race and education on MDE (Table 3).

Table 4 summarizes the results of two logistic regressions in the pooled sample, with MDE as the outcome and unemployment as the predictor. In *Model 1* that only included main effects, unemployment was not associated with odds of MDE in the pooled sample. *Model 2* that also included unemployment by gender and unemployment by race interactions did not show any interaction between gender and unemployment and race and unemployment on MDE (Table 4).

Table 5 summarizes the results of two logistic regressions in the pooled sample with MDE as the outcome and marital status as the predictor. In *Model 1* that only included main effects, marital status was not associated with lower odds of MDE in the pooled sample. *Model 2* that also included marital status by gender and marital status by race interactions did not show any interaction between gender and marital status and race and marital status on MDE (Table 5).

### 3.3. Multivariable Models on Separate Effect of Income across Race by Gender Groups

Table 6 summarizes the results of four logistic regressions, with MDE as the outcome and household income as the only SES indicator as the main predictor. High household income was protective for White women only (OR = 0.76, 95% CI = 0.64–0.91). High income was protective for White women only. High income was not associated with additional risk of MDE for African American men (OR = 1.05, 95% CI = 0.94–1.17) in the absence of other SES indicators.

Table 6 summarizes the results of four logistic regressions, with MDE as the outcome and all SES indicators as the predictors. High household income was protective for White women only. High education was (marginally significant) protective factor for African American women only. Different from the models that did not control for other SES indicators, high income became a (marginally significant) risk factor for African American men after controlling for the effects of all other SES indicators (Table 6).

## 4. Discussion

Our study revealed four major findings. First, race (African American) and high household income but not gender, education, employment, and marital status were protective against risk of 12-month MDE in the pooled sample. Second, income interacted with gender on MDE, suggesting a larger mental health gain from household income for women than men. Third, in race by gender specific models, high household income was protective for White women, and high education was protective for African American women. Forth, high income became a risk factor for African American men after controlling for the effects of other SES indicators.

The protective effect of SES indicators, such as income in the pooled sample is supported by an extensive theoretical and empirical work. For instance, Fundamental Cause Theory (FCT), developed by Link and Phelan (1995) [1,19], considers low SES as a fundamental cause of disease due to several features [19,20,21]. Multiple studies have shown that education and income are protective for a wide range of outcomes [9,10,11,12,13,14,15].

There are many mechanisms that explain why SES is linked to better mental health. High SES enables individuals to avoid risk factors and harmful exposures [1,19]. At the same time, SES minimizes consequences of risk when they are faced [20,21]. High SES such as education enhances population human capital [47], as well as health promoting behaviors, such as exercise and healthy diet. High SES reduces risk behaviors, such as smoking and substance use [48]. SES also enhances access to healthcare, through healthcare insurance coverage that follows employment. All of these changes are essential for healthy lives [1,19,20,21]. High SES also increases population access to materialistic resources, such as healthy food and safe living place [33]. High SES promotes human connections, and social network [34]. SES also increases psychosocial assets, such as sense of agency and mastery [49]. High SES is also a proxy of high rewarding low stress jobs that allow for more earnings and lower exposure to risk factors [50,51,52]. High SES is also shown to reduce the effects of a wide range of stressors including but not limited to economic hardship [47,53].

In the current study, White women’s mental health gained most from high household income. In a recent study using HRS data, White women, who were above age of 50, showed the most consistent protective effects of education and income against depressive symptoms, self-rated health (SRH), insomnia, physical inactivity, and body mass index (BMI). Most of these effects were, however, absent for African American men and women [54].

When other SES indicators were controlled, high income became a risk factor for MDE for African American men. Some of this finding was previously reported by Hudson et al. [36]. Using NSAL, they showed that household income was positively associated with the risk of MDE among African American men. For African American women, however, high income reduced the risk of 12-month MDE [36]. This study extends the previously reported findings by showing that high income does not start as a risk factor, but becomes a risk factor when other SES indicators are controlled. That means, high income is not per se a risk factor, but the residual effect of income beyond employment, marital status, and education becomes a risk factor for African American men. Using a national sample of African American boys, a recent study documented a positive association between income and MDE risk in African American boys [23]. Other studies have also suggested that high SES may be associated with an increased mental cost for African American men [35,54,55]. In the NSAL data, when the effects of religiosity and psychiatric disorders are controlled, more educated Black women reported a higher level of suicidality [35]. In the Americans Changing Lives (ACL) study, among African American men, high education credentials were a risk factor for an increase in depressive symptoms over a 25-year follow-up [55]. In the Health Retirement Survey (HRS), income did not protect African American men against sustained high BMI. High education attainment was negatively associated with better sleep quality, physical activity, and weight for all race by gender groups, with African American men being the only exception [54]. This literature is mixed and inconclusive [35,54,55].

Several mechanisms can explain the heterogenous effects of SES across social groups. How SES alters life circumstances of populations depends on the availability of other resources, which varies for Whites and African Americans [56]. SES resources, such as education and income, have differential effects on wealth, purchasing power, living conditions, life styles, and behaviors across race by gender groups [35,55,57,58,59]. For instance, the effects of education on drinking [57], depressive symptoms [55], suicidality [35], chronic disease [55], and mortality [58] varies for Whites and African Americans, in all cases, gain being smaller for the latter.

Very high costs of upward social mobility may potentially explain why high SES African American men are at risk of depression. Fuller Rowel and colleagues have shown that education attainment is associated with some social, psychological, and physiological costs among African Americans, particularly boys [33,34]. Another mechanism for the diminished return of SES for African American males is high costs of discrimination. For instance, Hudson et al. have shown that SES has the smallest health effects when discrimination is high, suggesting that exposure to racial discrimination may minimize the protective health effects of high SES among African American men [32]. This is particularly relevant to African American men, as men are more vulnerable to discrimination than women [60,61,62,63].

Our findings are against the traditional assumption that high SES is universally protective across populations. Growing evidence suggests that gender and race do not add, but interact with SES indicators in their impact on health [64,65]. In addition, it is not simply race or gender, but their intersection that modifies the patterns and the mechanisms by which SES protect health outcomes [65]. Rather than control variables, race and gender should be regarded as contextual factors that shape population susceptibility or resilience to risk and protective factors [17,18]. In statistical terms, race and gender should be conceptualized as effect modifiers rather than confounders [57]. Conceptually, these findings and arguments are in line with the intersectionality framework [63,66].

This is not the first report on race, gender, or race by gender variation in health gains due to SES. Everett et al. used the U.S. National Health Interview Survey data and showed significant differences over time in pattern, shape, and the magnitude of the gradient (linear) effects of education attainment on mortality across cohorts of White women and White men but not African American men and African American women [67].

Findings of this study should be interpreted in light of study limitations. First, this study only studied the intersection of race, gender, and SES; many other important variables, such as age, cohort, and place were not studied. Second, all of the study determinants were at the individual level, and the study did not include contextual and neighborhood level factors. Other covariates, such as health behaviors, including diet, exercise, and sleep were also not entered to this study [68]. Third, due to the cross-sectional design of our study, our findings do not indicate causation but association [69]. There is a need to replicate the current findings using longitudinal data [70,71]. Finally, validity of MDE is not identical in race by gender by class groups. Positive association between income and odds of MDE may be due to higher disclosure, lower social desirability, or higher mental health literacy of high SES African American men. Despite these limitations, this study is one of very few studies using a national sample to explore separate, additive, and multiplicative effects of race, gender, and class on MDE.

## 5. Conclusions

To conclude, the findings reported here suggest that the effects of race, gender, and SES on MDE are not additive but multiplicative. To be more specific, whether an SES indicator, such as income, operates as a risk or protective factor for MDE depends on race, gender, and other SES characteristics. Overall, for MDE as the outcome, White women are the only group who benefit from higher income and the residual effect of high income (above and beyond education, employment, and marital status) may become a risk factor for African American men.

## Figures and Tables

**Table 1:** Descriptive statistics in the pooled sample and based on race and gender.

Characteristics	All		Whites		African Americans	
	**Mean**	**95% CI**	**Mean**	**95% CI**	**Mean**	**95% CI**
Age	43.69	42.25–45.14	44.98	42.11–47.85	42.33	41.26–43.39
Education *	2.48	2.35–2.61	2.67	2.42–2.92	2.28	2.21–2.35
Income (1000USD) *	41.56	37.44–45.67	46.69	38.94–54.44	36.12	33.45–38.79
	**%**	**95% CI**	**%**	**95% CI**	**%**	**95% CI**
Region						
Northeast	19.73	13.89–27.25	23.08	12.61–38.41	16.12	14.16–18.30
Midwest	12.55	10.31–15.21	7.88	4.76–12.78	17.60	14.84–20.73
South	55.52	46.46–64.23	54.37	36.86–70.87	56.77	52.27–61.15
West	12.19	8.42–17.33	14.67	7.85–25.76	9.51	7.77–11.61
Socioeconomic status						
Education *						
11 Years or less	19.47	16.75–22.51	15.16	10.68–21.08	24.11	21.79–26.61
12 Years or more	80.53	77.49–83.25	84.84	78.92–89.32	75.89	73.39–78.21
Unemployment *						
Employed/Not In Labor Market	92.73	91.06–94.12	95.48	92.28–97.39	89.77	88.18–91.17
Unemployment	7.27	5.88–8.94	4.52	2.61–7.72	10.23	8.83–11.82
Marital Status *						
Married	51.78	47.94–55.61	54.19	46.83–61.38	41.77	39.64–43.92
Unmarried	48.22	44.39–52.06	45.81	38.62–53.17	58.23	56.08–60.36
Depression *						
No	92.68	91.76–93.52	92.12	90.46–93.51	93.30	92.29–94.18
Yes	7.32	6.48–8.24	7.88	6.49–9.54	6.70	5.82–7.71

* *p* < 0.05. CI: Confidence Interval.

**Table 2:** Logistic regression on the effects of income, race, and gender in the pooled sample.

	*Model 1* (Main Effects)		*Model 2* (Main Effects + Interactions)	
	OR	95% CI	OR	95% CI
Race (African Americans)	0.72 *	0.55–0.95	0.51 *	0.28–0.93
Gender (Women)	1.15	0.74–1.81	1.89 *	1.02–3.49
Age	0.99 ^#^	0.97–1.00	0.99	0.97–1.00
Region ^a^				
Midwest	0.97	0.58–1.60	0.98	0.59–1.62
South	0.53 ***	0.38–0.72	0.53 ***	0.39–0.74
West	0.41 *	0.18–0.93	0.39 *	0.18–0.88
Income (USD1000)	0.92 *	0.84–1.00	1.07	0.81–1.42
Income × Race	-	-	1.10	0.94–1.29
Income × Gender	-	-	0.87 *	0.76–1.00
Intercept	0.31 **	0.15–0.64	0.28 *	0.11–0.74

Source: National Survey of American Life-Adolescents Supplement (NSAL); Outcome: 12 Month Major Depressive Episode; OR odds ratio, CI confidence interval; ^#^ *p* < 0.1; * *p* < 0.05; ** *p* < 0.01; *** *p* < 0.001; ^a^ Reference category: Northeast.

**Table 3:** Logistic regression on the effects of education, race, and gender in the pooled sample.

	*Model 1* Main Effects		*Model 2* Main Effects + Interactions	
	OR	95% CI	OR	95% CI
Race (African Americans)	0.76 *	0.57–0.99	0.97	0.31–2.32
Gender (Women)	1.23	0.76–1.97	1.13	0.54–2.35
Age	0.99 ^#^	0.97–1.00	0.99 ^#^	0.97–1.00
Region ^a^				
Midwest	0.96	0.55–1.66	0.96	0.55–1.67
South	0.52 ***	0.37–0.73	0.52 ***	0.36–0.75
West	0.43 ^#^	0.18–1.03	0.42 ^#^	0.18–1.04
Education (12 Years or more)	0.72	0.44–1.19	0.81	0.20–3.20
Education × Race	-	-	0.72	0.25–2.05
Education × Gender	-	-	1.11	0.49–2.49
Intercept	0.29 **	0.13–0.65	0.26 *	0.70–0.98

Source: National Survey of American Life-Adolescents Supplement (NSAL); Outcome: 12 Month Major Depressive Episode; OR odds ratio, CI confidence interval; ^#^ *p* < 0.1; * *p* < 0.05; ** *p* < 0.01; *** *p* < 0.001; ^a^ Reference category: Northeast.

**Table 4:** Logistic regression on the effects of unemployment, race, and gender in the pooled sample.

	*Model 1* (Main Effects)		*Model 2* (Main Effects + Interactions)	
	OR	95% CI	OR	95% CI
Race (African Americans)	0.77 ^#^	0.58–1.02	0.77 ^#^	0.57–1.02
Gender (Women)	1.23	0.76–1.99	1.31	0.77–2.21
Age	0.99	0.97–1.00	0.99	0.97–1.00
Region ^a^				
Midwest	0.96	0.55–1.69	0.95	0.54–1.67
South	0.53 ***	0.37–0.76	0.52 ***	0.37–0.75
West	0.42 ^#^	0.17–1.05	0.42 ^#^	0.17–1.04
Unemployed	1.24	0.82–1.86	0.93	0.55–1.59
Unemployed × Race	-	-	2.03	0.71–5.81
Unemployed × Gender	-	-	1.00	1.00–1.00
Intercept	0.20 ***	0.11–0.38	0.20 ***	0.11–0.36

Source: National Survey of American Life-Adolescents Supplement (NSAL); Outcome: 12 Month Major Depressive Episode; OR odds ratio, CI confidence interval; ^#^ *p* < 0.1; * *p* < 0.05; ** *p* < 0.01; *** *p* < 0.001; ^a^ Reference category: Northeast.

**Table 5:** Logistic regression on the effects of marital status, race, and gender in the pooled sample.

	*Model 1* (Main Effects)		*Model 2* (Main Effects + Interactions)	
	OR	95% CI	OR	95% CI
Race (African Americans)	0.75 *	0.57–0.99	0.75 *	0.57–0.99
Gender (Women)	1.20	0.75–1.92	1.33	0.72–2.43
Age	0.99	0.97–1.00	0.99 ^#^	0.97–1.00
Region ^a^				
Midwest	0.99	0.56–1.74	0.98	0.54–1.76
South	0.55 **	0.36–0.84	0.55 **	0.35–0.86
West	0.42 ^#^	0.17–1.04	0.41 *	0.17–0.97
Married	0.74	0.43–1.28	0.67 ^#^	0.42–1.06
Married × Race	-	-	1.26	0.37–4.34
Married × Gender	-	-	1.00	
Intercept	0.23 ***	0.12–0.44	0.22 ***	0.10–0.50

Source: National Survey of American Life-Adolescents Supplement (NSAL); Outcome: 12 Month Major Depressive Episode; OR odds ratio, CI confidence interval; ^#^ *p* < 0.1; * *p* < 0.05; ** *p* < 0.01; *** *p* < 0.001; ^a^ Reference category: Northeast.

**Table 6:** Logistic regression on additive effects of socioeconomic resources based on race and gender.

	White Men		White Women		African American Men		African American Women	
	OR	95% CI	OR	95% CI	OR	95% CI	OR	95% CI
Age	1.00	0.96–1.04	0.98 ^#^	0.96–1.00	0.99	0.97–1.00	0.98 **	0.96–0.99
Region ^a^								
Midwest	0.59	0.08–4.15	0.94	0.07–13.22	3.99 *	1.15–13.87	0.83	0.49–1.42
South	0.24 *	0.06–0.95	1.06	0.38–2.93	1.29	0.49–3.39	0.45 ***	0.30–0.69
West	0.60	0.09–4.12	0.13 ***	0.05–0.32	1.90	0.43–8.43	0.80	0.43–1.47
Income (USD1000)	0.94	0.81–1.09	0.71 ***	0.59–0.86	1.10 ^#^	0.99–1.21	0.98	0.88–1.09
Education (12 Years or more)	0.98	0.23–4.25	1.64	0.69–3.89	0.60	0.29–1.25	0.69 ^#^	0.44–1.08
Unemployed	3.57	0.71–17.96	-	-	1.63	0.61–4.34	1.16	0.71–1.88
Married	1.59	0.27–9.51	1.33	0.67–2.64	0.35 *	0.14–0.92	0.70	0.43–1.14
Intercept	0.17	0.01–3.97	0.44	0.11–1.67	0.06 ***	0.02–0.24	0.50	0.21–1.19

Source: National Survey of American Life-Adolescents Supplement (NSAL); Outcome: 12 Month Major Depressive Episode; OR odds ratio, CI confidence interval; ^#^ *p* < 0.1; * *p* < 0.05; ** *p* < 0.01; *** *p* < 0.001; ^a^ Reference category: Northeast.
